# A quantitative infection assay for human type I, II, and III interferon antiviral activities

**DOI:** 10.1186/1743-422X-10-224

**Published:** 2013-07-06

**Authors:** Emily Voigt, Bahar İnankur, Ashley Baltes, John Yin

**Affiliations:** 1 Department of Chemical and Biological Engineering, University of Wisconsin, Madison, USA; 2 Systems Biology Theme, Wisconsin Institute for Discovery, 330 N Orchard St., Madison, WI 53715, USA

**Keywords:** Antiviral activity assay, Reporter virus, Interferon, Paracrine signaling, Bioassay, Cytokine quantification

## Abstract

**Background:**

Upon virus infection, cells secrete a diverse group of antiviral molecules that signal proximal cells to enter into an antiviral state, slowing or preventing viral spread. These paracrine signaling molecules can work synergistically, so measurement of any one antiviral molecule does not reflect the total antiviral activity of the system.

**Results:**

We have developed an antiviral assay based on replication inhibition of an engineered fluorescent vesicular stomatitis virus reporter strain on A549 human lung epithelial cells. Our assay provides a quantitative functional readout of human type I, II, and III interferon activities, and it provides better sensitivity, intra-, and inter-assay reproducibility than the traditional crystal violet based assay. Further, it eliminates cell fixation, rinsing, and staining steps, and is inexpensive to implement.

**Conclusions:**

A dsRed2-strain of vesicular stomatitis virus that is sensitive to type I, II, and III interferons was used to develop a convenient and sensitive assay for interferon antiviral activity. We demonstrate use of the assay to quantify the kinetics of paracrine antiviral signaling from human prostate cancer (PC3) cells in response to viral infection. The assay is applicable to high-throughput screening for anti-viral compounds as well as basic studies of cellular antiviral signaling.

## Background

Mammalian cells respond to virus infection by synthesizing and secreting a host of antiviral molecules that are not only involved in recruitment of immune effector cells and activation of adaptive immunity, but also control localized spread of virus infection via mechanisms of “cell-intrinsic” innate immunity. These secreted antiviral molecules, first described collectively in 1957 as “interferon”, establish an anti-viral state within the infected cell, and signal other cells to react in an antiviral manner [1,2].

Antivirals secreted by non-immune cells comprise a diverse mixture of molecules which exert a combined paracrine effect on proximal cells. These include, for example, the classic type I interferons (IFNα/β, etc.) [3,4], type II interferon (IFNγ), and type III interferons (IFNλs) [5-9]. While type I interferons are known to play important roles in host antiviral responses, for many viruses such as human rhinovirus and influenza infections of bronchial epithelial cells, other interferons such as the more recently identified IFNλs, may dominate [10,11]. Thus, measuring type I IFNs alone does not necessarily accurately assess cellular antiviral responses, and an assay that measures antiviral responses due to multiple types of interferons is required.

The biological effects of interferons are often measured using reporter gene assays (RGAs), which use transgenic cell lines expressing a reporter gene driven by an IFN-responsive promoter [12-17]. These assays accurately measure the presence or upregulation of single molecules, such as Mx or various interferon-stimulated genes, and provide valuable information on specific components of an antiviral response. However, other assay methods are necessary for the quantification of the integrated antiviral effects of multiple-type interferon signaling, which is essential for studying inhibition of virus spread [18,19]. Moreover, functional measures of secreted antiviral signaling will be useful for advancing experimental and computational models of virus-cell interactions and viral infection spread in monolayers and tissues [20-24]. Such an assay potentially has additional applications in the area of high-throughput screening for antiviral compounds.

The most traditional form of a functional antiviral assay is the assay based on cytopathic effect (CPE), commonly used to determine the potency of purified interferon stocks. In the CPE assay, antiviral activity is measured based on its ability to inhibit virus-induced cytopathology as measured by a crystal violet live-cell stain [25]. While widely used, these types of assays are labor-intensive and contain many handling steps that can disturb cell layers and increase variability. Additionally, successful infection that does not cause major cytopathology is not detected by these assays. These shortcomings can be addressed through the development of reporter virus strains as robust readouts of virus replication. Examples include a luciferase-expressing reporter strain of the BSL3 Rift Valley Fever virus and a non-proliferative vesicular stomatitis virus (VSV) replicon also expressing luciferase [2,26]. Here we create a replication-competent fluorescent reporter VSV strain. We use this virus in a simple, sensitive, and reproducible assay for detection of secreted antiviral signaling activity. The assay does not require the addition of expensive substrates eliminates cell fixing, rinsing, and staining steps, and significantly improves sensitivity, and reproducibility over the traditional crystal violet CPE assay. Additionally, the broad tropism of VSV allows for potential future assay adaptation to a large range of host cell types [2,26].

## Results

### Synthesis of reporter virus constructs

**Figure 1:**
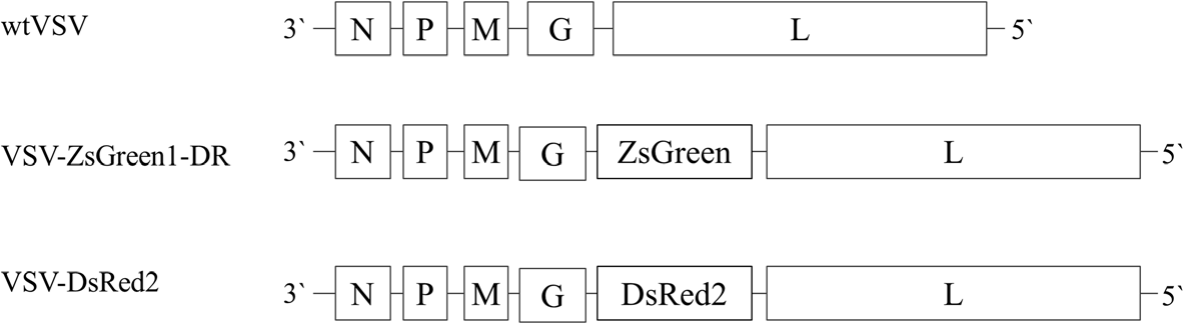
**Genotype maps of wild-type and fluorescent reporter VSV strains used in this study.** Recombinant VSV strains were created by reverse-genetics, incorporating fluorescent reporter variants of GFP(ZsGreen) and RFP(DsRed2) along with the five native VSV proteins: nucleocapsid protein (N), phosphoprotein (P), matrix protein (M), glycoprotein (G), and large protein (L).

We sought to create an assay to quantify overall activity of secreted antiviral molecules with optimal sensitivity and reproducibility. We therefore compared four detection methods to determine antiviral activity: the traditional crystal violet live-cell stain, Sytox® orange, a fluorescent dead cell stain, and both a DsRed2-expressing and a ZsGreen-expressing VSV reporter strain. Using fluorescent reporters of VSV replication may increase assay sensitivity, as inhibition of virus replication is a more direct measure of antiviral activity than cytopathology alone. We created strains of DsRed2- or ZsGreen-encoding VSV with genomic organization as indicated in Figure 1 for use in our antiviral assays. We tested the ability of these recombinant virus strains to infect A549 lung epithelial cells, and found their replication rates comparable to that of their non-fluorescent equivalent (Additional file 1: Figure S1) and a multiplicity of infection (MOI) of 5 pfu/cell sufficient to homogenously infect A549 monolayers (data not shown).

### Comparison of assay readout methods

**Figure 2:**
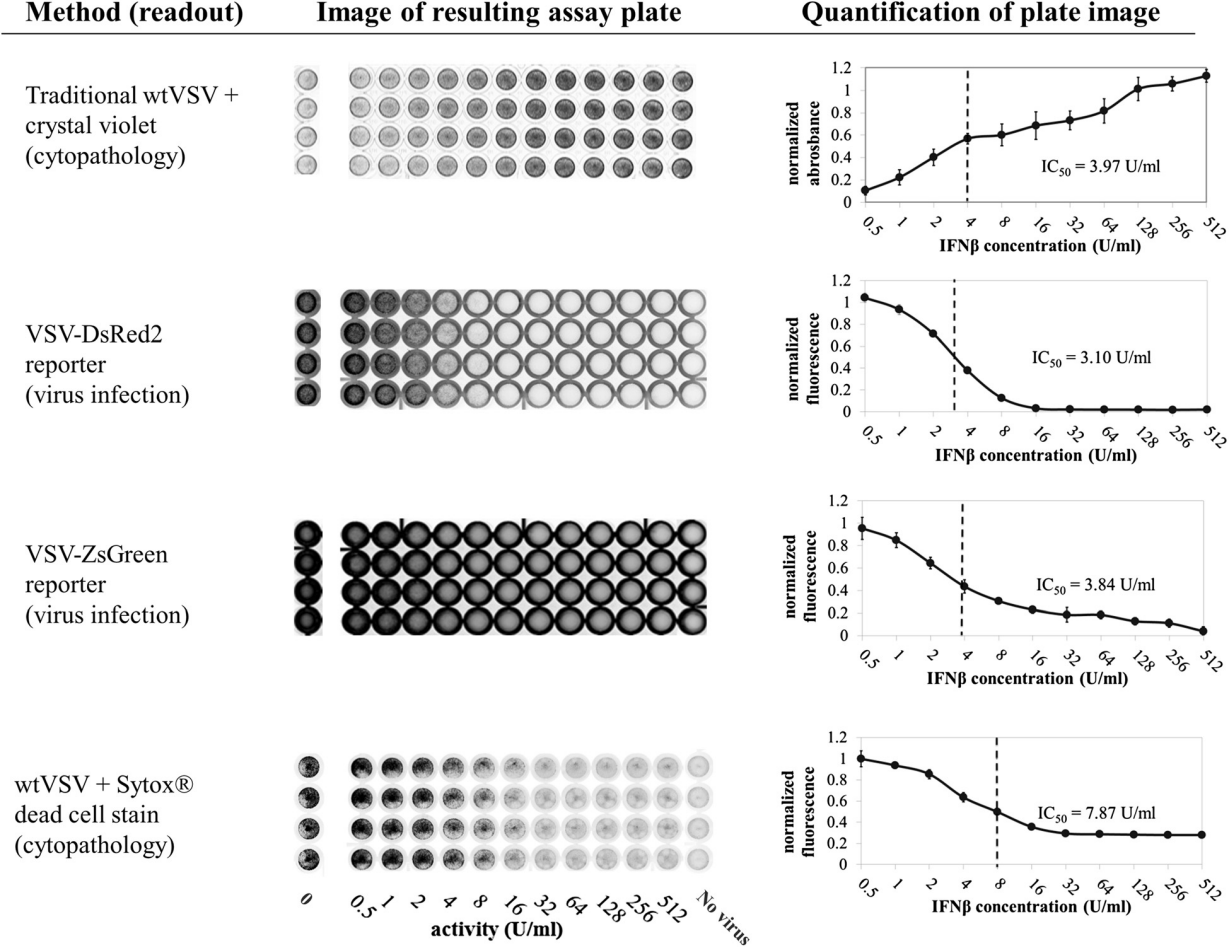
**Comparison of live/dead cell stains and fluorescent-protein expressing assay virus.** A549 cells were incubated under serial 2-fold dilutions of recombinant human IFNβ for 24 hours, then infected with either wild-type or recombinant VSV as indicated at a multiplicity of 5 pfu/cell. After 24 hours of infection, assay plates were stained, imaged, and quantified as discussed in the Methods section. Positive signal indicates cell survival for the crystal violet assay, virus replication for the fluorescent virus assays, and cell death in the Sytox assay. Positive control wells are cells untreated by antivirals and infected. Negative control wells are untreated, uninfected cells.

We compared the traditional virus-induced cytopathology measurement (crystal violet after 28-hour infection) with a more recently developed fluorescent dead cell stain (Sytox®) and our two fluorescent VSV reporter strains. To do so, we incubated A549 cells under 2-fold dilutions of an IFNβ standard solution in media for 24 hours. The cells were then infected with either wild-type or one of the fluorescent reporter VSV strains as indicated in Figure 2, at an MOI of 5 pfu/cell, and the infection was allowed to progress for 28 hours. WtVSV-infected plates were rinsed and stained as indicated. Assay results were quantified by fluorescent scanning at the appropriate wavelengths and subsequent normalization to positive (no IFN) and negative (no virus) controls, as shown in Figure 2.

We found that the fluorescent signal from DsRed2 and ZsGreen reporter viruses created reproducible gradients indicating decreasing viral replication due to increasing IFN concentrations. These gradients were comparable to those found by staining cells with crystal violet after wtVSV replication and cell death. Sytox® fluorescent dead cell stain also produced quantifiable gradients, but with a significantly poorer antiviral detection limit (IC_50_ = 7.87 U/ml vs. <4 U/ml) than both crystal violet and fluorescent virus assays.

### Sensitivity and reproducibility comparison of fluorescent reporter viruses

As fluorescent signals are generally more sensitive than readouts of absorbance, and dead-cell stains require fixing and staining steps that can disturb cell layers and increase variability, we tested the reproducibility and sensitivity of assays using ZsGreen and DsRed2 reporter virus and compared it to that of the traditional crystal violet cytopathology-based assay. Fluorescence was read both on a standard fluorescent plate reader and also scanned with a high-resolution GE Typhoon FLA 9000 Biomolecular Imager, to determine which reading method is more sensitive and reproducible. Sensitivity was defined as the limit of detection (LOD), the lowest antiviral starting dose that, upon two-fold serial dilution, would produce a dose–response curve crossing the 50% viral inhibition point and allow for accurate IC_50_ determination, as shown in Table 1. Calculation of IC_50_ values for both quantification types was done using linear interpolation within the linear dose-dependent range of the indicated interferon treatment as described in Methods. A sigmoidal-fit IC_50_ calculation method was also tested, but showed no advantage over linear interpolation.

**Table 1:** Assay statistics and reproducibility

***A. Fluorescent plate scanner and whole-well signal integration***							
		**Sensitivity (LOD) units/ml**	***Variability intra-assay COV (%)***				***Variability inter-assay COV (%)***
			**Assay1**	**Assay 2**	**Assay 3**	**Assay 4**	
IFNα	VSV-DsRed2	1.51 +/− 0.12	0.9	2.9	0.7	1.7	2.4
	VSV-ZsGreen	2.07 +/− 0.94	1.8	8.6	5.5	3.3	16.0
IFNβ	VSV-DsRed2	1.71 +/− 0.27	1.4	1.1	0.6	1.0	5.1
	VSV-ZsGreen	2.57 +/− 0.52	1.9	4.1	2.2	3.2	7.9
***B. Fluorescence/absorbance plate reader***							
		**Sensitivity (LOD) units/ml**	***Variability intra-assay COV (%)***				***Variability inter-assay COV (%)***
			**Assay 1**	**Assay 2**	**Assay 3**	**Assay 4**	
	VSV-DsRed2	1.29 +/− 0.34	4.0	1.7	1.9	5.7	5.7
IFNα	VSV-ZsGreen	0.96 +/−0.18	1.2	1.1	1.7	2.5	3.6
	Crystal violet	4.09 +/− 0.43	17.5	14.0	7.3	8.6	21.9
	VSV-DsRed2	2.71 +/− 0.29	3.0	1.8	1.7	3.6	6.4
IFNβ	VSV-ZsGreen	1.87 +/− 0.11	2.3	3.1	0.7	1.8	4.4
	Crystal violet	8.44 +/− 2.28	20.1	6.6	4.3	7.3	18.5

Viral replication reporters and two methods of signal measurement were tested for the functional antiviral assays and calculated IC_50_ values statistically compared with the traditional crystal violet antiviral assay. Coefficients of variance were calculated with a minimum of four replicates. +/− values are 95% confidence intervals over all samples.

**Figure 3:**
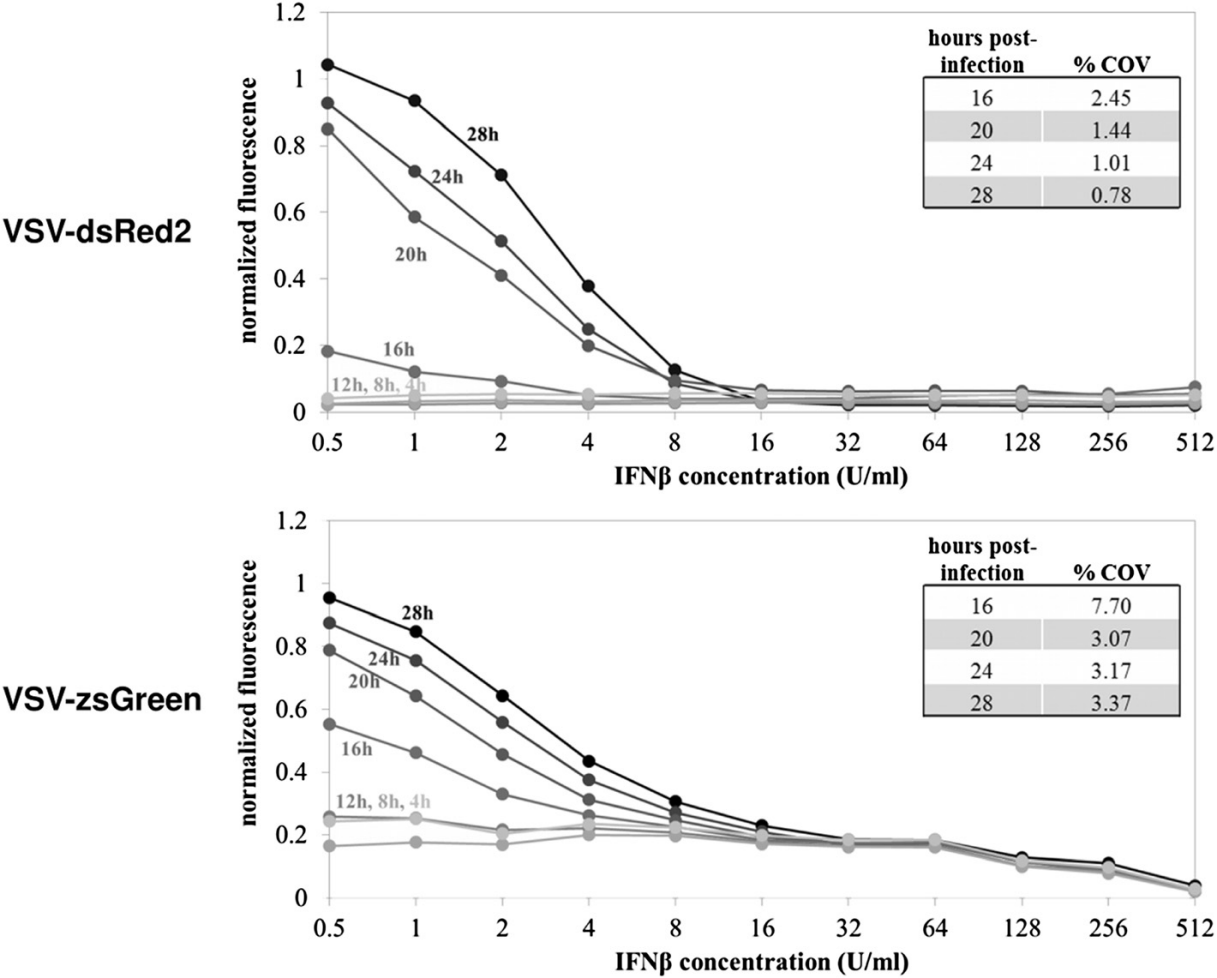
**Assay time-course development.** Recombinant VSV antiviral activity assays were imaged on a fluorescent biomolecular imager every four hours post-infection to monitor fluorescent signal development indicating viral replication. Mean fluorescence values were extracted from plate images, normalized to positive and negative controls and plotted. Darker markers indicate longer development times. IC_50_ values were calculated as described, and the coefficient of variance was calculated between four replicates at each time point.

Both fluorescent reporter viruses significantly decreased the limit of detection and improved intra- and inter-assay reproducibility over the traditional crystal violet staining method. Sensitivity differences between DsRed2 and ZsGreen were insignificant (p > 0.1). The DsRed reporter virus showed better inter-assay reproducibility than the ZsGreen virus when quantified by fluorescent scanning. This may be due to fluorescent background in the green absorption/emission spectrum produced by the plastic microtiter plate (Figure 3). There was no significant difference between the reporter viruses for the plate reader quantification (p-value = 0.44).

### Optimization of fluorescent signal

We determined the optimal time for signal development by measuring a time course over 28 hours of signal development as shown in Figure 3. As time progresses, the dose-dependent signal gradient develops, where higher fluorescent intensity was observed at low interferon concentrations where reporter virus replicates more productively. We found that a gradient sufficient for IC_50_ calculation first develops for the DsRed2 virus 16 hours post-infection. The coefficient of variance decreases until 28 hours post-infection, but met our goal of 1% coefficient of variance as early as 24 hours post-infection. This gradient shows a linear range between 0.5 and 16 units/ml. The minimum measurable IC_50_ for each set of conditions was between 1–3 units/ml for IFNβ, comparable to other published assays [2,6,12,14-17]. The coefficient of variation for the ZsGreen IC_50_ improves until 20 hours, then levels off but remains considerably higher than for the VSV-DsRed2 assay. From these results, the VSV-DsRed2 assay developed for 24–28 hours appears optimal, providing excellent sensitivity, reproducibility and lowest background. We chose a development time of 24 hours as our standard for convenience. Our final assay procedure and comparison to the traditional crystal violet assay is shown in Table 2.

**Table 2:** Comparison of infection reporter assay and the traditional crystal violet antiviral assay

	**Crystal violet assay**	**VSV-DsRed2 assay**
Antiviral incubation	24 hours, 67ul sample	24 hrs, 67ul sample
Infection	wt virus, 24 hour incubation	RFP reporter virus, 24 hour incubation
Fixation	4% PFA in 5% sucrose, 20 min	
Rinse	2x PBS	
Stain	Addition of crystal violet, overnight incubation	
Scan	Microplate reader at 570 nm	Fluorescent microplate reader 485/620 or fluorescent scanner 555/580 nm.

### Validation of assay against human type I, II, and III interferons

**Figure 4:**
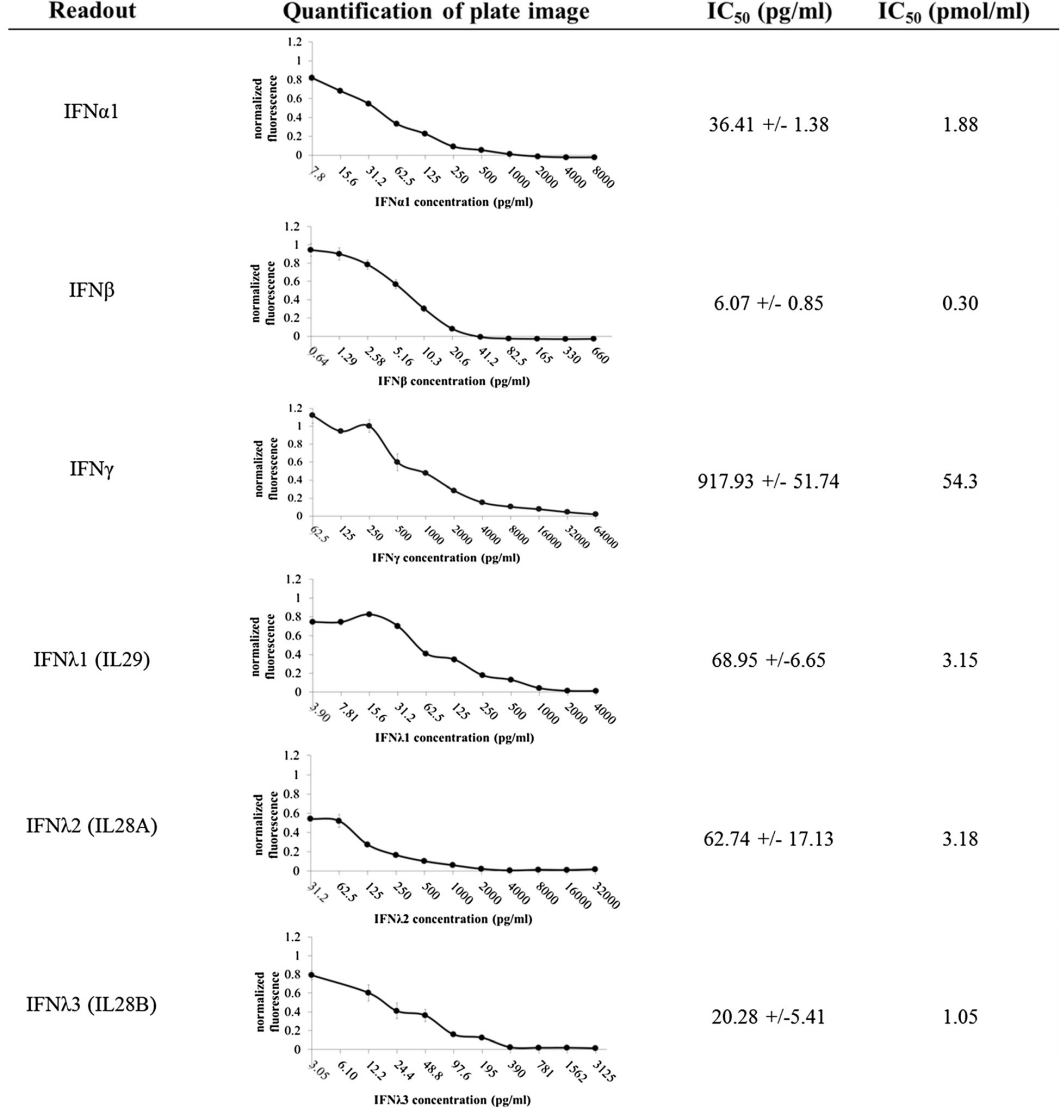
**Validation of the assay’s ability to measure multiple interferon types.** The final DsRed2 form of the assay was used to measure antiviral activity of human type I (IFNα1, IFNβ), type II (IFNγ), and type III (IFNλ1, IFNλ2, IFNλ3) recombinant bioactive interferon standards. IC_50_ values in pg/ml of added interferon were calculated as described, and are also shown in pmol/ml for molar comparisons.

We tested the ability of the assay to detect antiviral activity of several human type I, II, and III interferons, using the final assay method as described in Table 2. Recombinant human interferons α1 and β were used to represent type I IFNs, IFNγ was tested as the sole type II IFN, and interferons λ1 (IL-29), λ2 (IL-28A) and λ3 (IL-28B) represented type III IFNs. Antiviral activity was successfully detected and measured from all IFN samples using our assay, as shown in Figure 4. IC_50_ concentrations were lowest for IFNβ, and highest for IFNγ, as expected. As the antiviral effects of IFNγ are largely due to cell-mediated and adaptive immune responses absent in our assay, we measured only the direct antiviral effects of IFNγ which are far less potent than the type I and III interferons.

### Demonstration of the assay to measure antivirals produced in response to VSV infection

**Figure 5:**
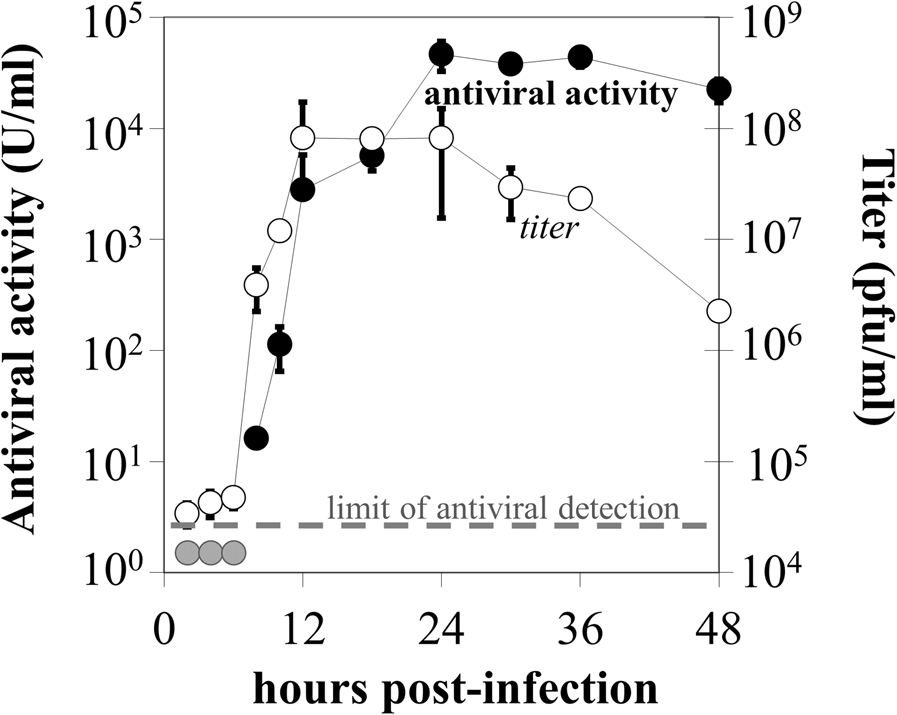
**Kinetics of functional antiviral signaling and virus progeny release of PC3 cells in response to virus infection.** Data points are averages of biological duplicates assayed in duplicate (titer) or quadruplicate (activity). Three antiviral activity data points (2, 4, and 6 hours post-infection) resulted in antiviral signal beneath the assay detection limit (grey). Closed symbols: antiviral activity. Open symbols: viral titer. Error bars are +/− standard deviation.

Finally, we tested the suitability of our assay for measuring the kinetics of secretion of antiviral factors by another human cell type infected with virus. We infected parallel wells of prostate cancer (PC3) cells at MOI 50 with M51R-mutant VSV, a strain that is attenuated in its ability to block the cellular antiviral response to infection [27]. Supernatants were sampled from parallel wells over the course of infection. The titer of the supernatant samples was measured by plaque assay. Separate aliquots of supernatant were UV-irradiated to deactivate live virus, serially diluted, and assayed (Figure 5).

Extracellular antiviral activity was first detected between 6 and 8 hours post-infection (hpi), rapidly rising from 8 to 12 hpi, concurrent with the release of virus progeny. The antiviral activity continued to increase at a slower rate from 12 to 24 hpi before reaching a plateau. The range of activity, which spanned four orders of magnitude, represents the collective effects of all secreted and extracellular factors that trigger antiviral responses in A549 cells, independent of their specific pathways. This highlights the ability of this method to capture an integrated picture of the antiviral response.

## Discussion

We have developed a functional antiviral assay, based on the inhibition of fluorescence produced during infection by an engineered RFP reporter strain of vesicular stomatitis virus, which can be used to report the combined antiviral activity of human interferons α, β, γ, λ1, λ2, and λ3. The assay shows an improvement in reproducibility over most published assays, including more recently published assays using luciferase reporter viruses or cells. Sensitivity of the assay, as defined as the low limit of detection, is also comparable to luciferase-based antiviral assays. However, the linear range of luciferase assays has a higher saturation concentration than the assay presented here, so further dilution of antiviral sample may sometimes be necessary when using this method (see Additional file 2: Table S1 for assay comparisons). The fluorescent reporter virus used in this assay is also easily propagated on standard laboratory cell lines, as opposed to non-replicative particles [28], leading to cheaper, more renewable and readily available assay reagents. Additionally, the assay avoids the use of luciferase reagents [2,12,14,28] which increase assay expense, and eliminates many handling steps which can introduce variability and error.

VSV is highly sensitive to interferon signaling, making it a good choice to assay for antiviral activities. However, if the ability of antiviral signaling molecules to inhibit specific viruses is of interest, or signaling in other tissue types is to be more closely investigated, this assay could likely be adapted for a multitude of virus/cell combinations. Fluorescent-expressing viruses are common throughout virological research, allowing this method to be adapted with a minimum of reagent development. Additionally, the assay could potentially be usable for measuring antiviral paracrine signals from samples obtained *in vivo*, such as irradiated serum and nasal lavage. Due to its ease and low expense, it could also be applied to the high-throughput screening of small molecules for antiviral properties.

In contrast to most other published assays, our assay is non-specific to a particular signaling pathway or network, providing a measure that is complementary to pathway-specific assays using bioengineered reporter cells [12,14,16]. The assay can thus be used to detect the collective effects of antiviral secreted factors from various antiviral pathways. We note, however, that since antiviral responses can be cell-type dependent, the use of A549 cells makes this assay most suitable for studies of antiviral secretions from respiratory cells. As such, our assay is best suited for studies of early cellular responses to upper respiratory tract infections, such as influenza A, respiratory syncytial virus, and some rhinoviruses. Additionally, our assay is not limited to quantifying the antiviral activity of interferons. Other secreted potentially antiviral molecules include interferon-stimulated gene 15 (ISG15) [29-31], inflammatory factors such as TNFα and IL-1β [32-34], and other species with antiviral function such as various interleukins [35-37], interferon gamma-induced protein 10 (IP-10), and antiviral microRNAs [38-40]. While we have not specifically tested the ability of our assay to detect these molecules, they may well contribute to the antiviral signal reported by our assay when testing cellular or tissue responses to infection.

Extrapolation and application of any quantitative, kinetic results obtained in cultured transformed cells to an *in vivo* system should only be done with discretion. However, characterizing the paracrine antiviral responses of cultured cells and the integrated effects of the multiple types of released interferons is important to better understand the complex interaction of antiviral signaling and virus spread throughout more intricate tissue systems.

## Conclusions

The assay presented here provides a functional measurement of antiviral activity of recombinantly expressed or cellullarly secreted human type I, III, and III interferons. It quantifies the potentially synergistic combination of antiviral activities due to multiple types of interferons in a biological sample, and is therefore complementary to pathway-specific measures of cell responses.

## Methods

### Cell culture

Human lung epithelial carcinoma (A549, ATCC CCL-185) and human prostate cancer (PC3, ATCC CRL-1435) cells were obtained from American Type Culture Collection and grown in RPMI 1640 medium (Gibco®) supplemented with 10% fetal bovine serum (FBS) (Atlanta Biologicals, Lawrenceville, GA). Baby hamster kidney (BHK-21) cells for plaque assays, originally obtained from Isabel Novella (University of Toledo), were grown in minimal essential medium (MEM, Corning) with 10% FBS and 2 mM Glutamax I (Gibco®). All cell lines were cultured in a humidified incubator at 37°C in 5% CO_2_. Cells lines were tested for mycoplasma contamination on a monthly basis.

### Cytokines and reagents

Universal type I interferon (human interferon alpha A/D, IFNα) and recombinant human IFN beta 1a (IFNβ) were purchased from PBL InterferonSource (Piscataway, NJ). Interferon antiviral activity levels in units/ml were confirmed by comparison with NIH standard Human Interferon Beta (NR-3080) as obtained from BEI Resources using the traditional interferon activity assay (VSV/A549) as described below. Sytox® Orange nucleic acid dead cell stain was obtained as a 5 mM solution in DMSO from Invitrogen and used at a final concentration of 0.25 μM. Crystal violet was obtained from PML Microbiologicals.

Recombinant human interferons alpha 1a, gamma, lambda 1, lambda 2, and lambda 3 were obtained from Cell Signaling Technology (Danvers, MA).

All experimental research in this work was done under the oversight of the University of Wisconsin Institutional Biosafety Committee and Office of Biological Safety. No human or animal subjects were used.

### Virus strains

Fluorescent VSV reporter virus strains incorporating either ZsGreen or DsRed2 into the fifth genomic position of VSV-Indiana were created using published reverse genetics techniques [41,42]. Adapted plasmids pBS-N, pBS-P, pBS-L, and pVSVFL(+) [42], for the expression of VSV N, P, and L genes and antigenomic VSV RNA under a T7 promoter were generously provided by Dr. Valery Grdzelishvili [43]. ZsGreen1-DR and DsRed2 genes (Clontech, Mountain View, CA) were PCR-amplified with the following primers and inserted into plasmid pVSVFL(+) in the fifth gene position:

ZsGreen For 5′-aactcaaatcctg*tatgaaaaaaactaacagatatccgtacg*gccaccatggcccagtcc-3′,

DsRed2 For 5′-aactcaaatcctg*tatgaaaaaaactaacagatatccgtacg*gccaccatggcctcctcc-3′,

ZsGreen Rev 5′-gaagaatctggctaggagtcgcggccgcctacaca-3′, and

DsRed2 Rev 5′-gaagaatctggctagcgctacaggaacaggtggtgg-3′.

These primers incorporated an overlap with plasmid pVSVFL(+) digested with NheI (overlap underlined) for In-Fusion Cloning (Clontech) as well as an additional VSV transcription unit (italicized) [44]. Successful insertion of the fluorescent protein genes into plasmid pVSVFL(+) was confirmed via Sanger sequencing.

In addition to the fluorescent VSV reporter strains, a recombinant VSV strain with a well-studied mutation to the M protein was created. This methionine to arginine substitution at the 51^st^ amino acid abolishes the ability of VSV M protein to inhibit host cell gene expression [45,46]. The M51R mutation was introduced to the M protein region of the genome via multistep PCR site-directed mutagenesis with the following primers (mutation in bold).

XbaI For 5′-ttgttctcatctagaggagagttcatctctgtcggaggtgac-3′

M51R Rev 5′-attcggatcataggtgtcc**c**tctcgtcaactccaaa-3′

M51R For 5′-tttggagttgacgaga**g**ggacacctatgatccgaat-3′

NheI Rev 5′-gaagaatctggctagcaggatttgagttactttccaagtcgg-3′

PCR reaction A used primers XbaI For and M51R Rev, and PCR reaction B used primers M51R For and NheI Rev. These PCR reactions both created fragments with the desired mutation, and because primers M51R For and M51R Rev are reverse compliments of each other, the products from PCR reactions A and B overlap for 36 bases. Products A and B were mixed and used as templates with primers XbaI For and NheI Rev in a third PCR reaction to produce a DNA fragment that spanned between two unique restriction enzyme sites and contained the desired M51R mutation. This fragment was then cloned into pVSVFL(+) digested with XbaI and NheI.

The presence of the desired mutation was confirmed in the plasmid and recovered infectious VSV via Sanger sequencing.

Infectious VSV was recovered from plasmid with T7 expressing vaccinia virus (VVT7), also from Valery Grdzelishvili, on BHK cells at 36°C as previously described [41,42,47]. Recovered VSV was separated from VVT7 via filtration with a 0.22 μm Millex GV filter unit (Millipore, Billerica, MA), amplified for 2 days on BHK cells, and plaque purified. A master and subsequent working stock of recovered recombinant VSV were created from a single plaque. Growth curves confirmed the recombinant VSV strains to have similar growth rates as recombinant wtVSV (Additional file 1: Figure S1).

### Antiviral activity assay

67 μl/well of A549 cells were seeded into 96-well microtiter plates at a density of 2.5×10^5^ cells/ml and cultured for 24 h before antiviral treatment. Interferon was diluted serially 1:2 in RPMI media supplemented with 2% FBS to final concentrations of 512 U/ml to 0.5 U/ml using an epMotion 5070 automated pipetting system. Culture media was vacuum aspirated from 96-well plates with confluent cell monolayers, 67 μl/well of antiviral dilution or control media was added, and plates were again incubated under culture conditions for 24 hours. After 24-hour incubation, cells were challenged with virus (wtVSV, VSV-ZsGreen or VSV-DsRed2, as indicated) in 30 μl RPMI media + 2% FBS per well added to the antiviral dilution for a final multiplicity of infection (MOI) of 5 pfu/cell.

In the standard antiviral assay with wtVSV infection, the infection was allowed to progress until cytopathic effects were readily apparent in unprotected control cells (16–28 hpi, as indicated). The cell medium was discarded, and cells were fixed with a solution of 4% paraformaldehyde (w/v) and 5% sucrose (w/v) in PBS for 20 minutes. The cells were rinsed twice with PBS (Sigma) and stained with crystal violet (0.1% w/v) in 20% ethanol overnight.

Alternatively, wtVSV-infected, unfixed assay plates were treated with fluorescent dead cell stain (Sytox® Orange, Invitrogen) 28 hours post-infection as an endpoint fluorescent readout of cell pathology. Fluorescent virus replication was measured without stain or fixation.

### Imaging

Crystal violet staining was measured with a Synergy H4 hybrid multi-mode microplate reader (BioTek) reading absorbance at 570 nm, and scanned using a desktop scanner to obtain reference images. Sytox® Orange, ZsGreen, and DsRed2 were detected by the microplate reader in fluorescence mode (485/620, 485/528, and 485/620, respectively). All fluorescent assay plates were also scanned with a GE Typhoon FLA 9000 Biomolecular Imager (ZsGreen 489/508 nm, DsRed2 555/580 nm, Sytox® Orange 555/580 nm) under BSL 2 conditions.

### Image quantification and analysis

Fluorescent scanning images were analyzed by using JEX, a customized JAVA-based batch processing image analysis platform incorporating much of the functionality of Image J (Rasband, 1997–2012) that can be found as shareware at <http://sourceforge.net/projects/jextools>. The mean fluorescent intensity of each well was extracted using JEX. Data for all assays were scaled using the following formula:

$$\frac{\;\mathrm{Sample}\;\mathrm{read}–\mathrm{average}\;\mathrm{of}\;\mathrm{uninfected}\;\mathrm{control}\;\mathrm{reads}}{\mathrm{Average}\;\mathrm{of}\;\mathrm{untreated},\mathrm{infected}\;\mathrm{controls}–\mathrm{average}\;\mathrm{of}\;\mathrm{uninfected}\;\mathrm{control}\;\mathrm{reads}}$$

IC_50_ value calculations for each dilution series were found by linear least-squares regression through the three data points in the linear range of the dose–response curves closest to half-maximum intensity. Subsequent interpolation determined the standard interferon dilution corresponding to a 50% decrease in signal above background with respect to the positive (infected, untreated) and negative (uninfected, untreated) control wells. The limit of detection was defined as the minimum interferon concentration that resulted in an IC_50_ curve that included the 50% viral inhibition point.

### Statistical analysis

For assay development the antiviral activity of interferon samples were tested in quadruplicate. The intra-assay coefficient of variance (COV) was calculated using the average of the quadruplicate IC_50_ values and their standard deviation. Inter-assay COV was calculated using average data from four separate assays and the standard deviation thereof. Comparisons between data sets were conducted using a two-tailed Student’s t-test assuming unequal sample variances.

### One-step virus infection

2 ml/well of PC3 cells were seeded in 6-well plates at a density of 2.5 10^5^ cells/ml and cultured for 24 h in RPMI supplemented with 10% FBS, until cells formed 70-90% confluent monolayers. Cells were then infected with mutant VSV (M51R) at a multiplicity of 5 in 200 μl RPMI with 2% FBS, and cells were incubated at 37°C for 1 h to allow for adsorption with rocking at 20 minute intervals. Mock-infected controls were incubated under 200 μl of RPMI media with 2% FBS. All cells were then rinsed with PBS once to remove unbound virus and 2 ml of RPMI with 2% FBS was added. Infection was allowed to progress under standard culture conditions. At the indicated times post-infection measured from the initial point of virus addition, supernatants were removed from cells and stored at −80°C. The experiment was conducted with full biological duplicates for every sample.

Virus in 400 μl of each infection supernatant sample was inactivated by exposure to 7000 J/m^2^ UVC irradiation in standard 24-well tissue culture plates with rocking over 20 minutes. Infection supernatants and corresponding controls were serially diluted in RPMI media with 2% FBS and antiviral activity was quantified, with technical duplicates, using the antiviral activity assay with the DsRed2-VSV reporter of viral replication. Virus titers from each sample were quantified prior to irradiation using standard plaque assays on BHK monolayers.

## Competing interests

The authors declare that they have no competing interests.

## Authors’ contributions

EV and Bİ carried out the antiviral assays. AB generated the recombinant virus strains. EV, Bİ, and JY designed the studies and were responsible for drafting and finalizing the manuscript. All authors read and approved the manuscript.

## Supplementary Material

Additional file 1: Figure S1 Kinetics of VSV strain growth on A549 cells. A549 cells were infected in parallel wells, MOI = 10, and parallel supernatant samples were taken over time and titered by plaque assay. Click here for file

Additional file 2: Table S1 A brief comparison of several published antiviral assays. Click here for file
