# Supplementary figures and images for: A quantitative infection assay for human type I, II, and III interferon antiviral activities
Source: Virol J. 2013 Jul 6;10:224. doi: 10.1186/1743-422X-10-224 (PMC3716869; doi:10.1186/1743-422X-10-224)

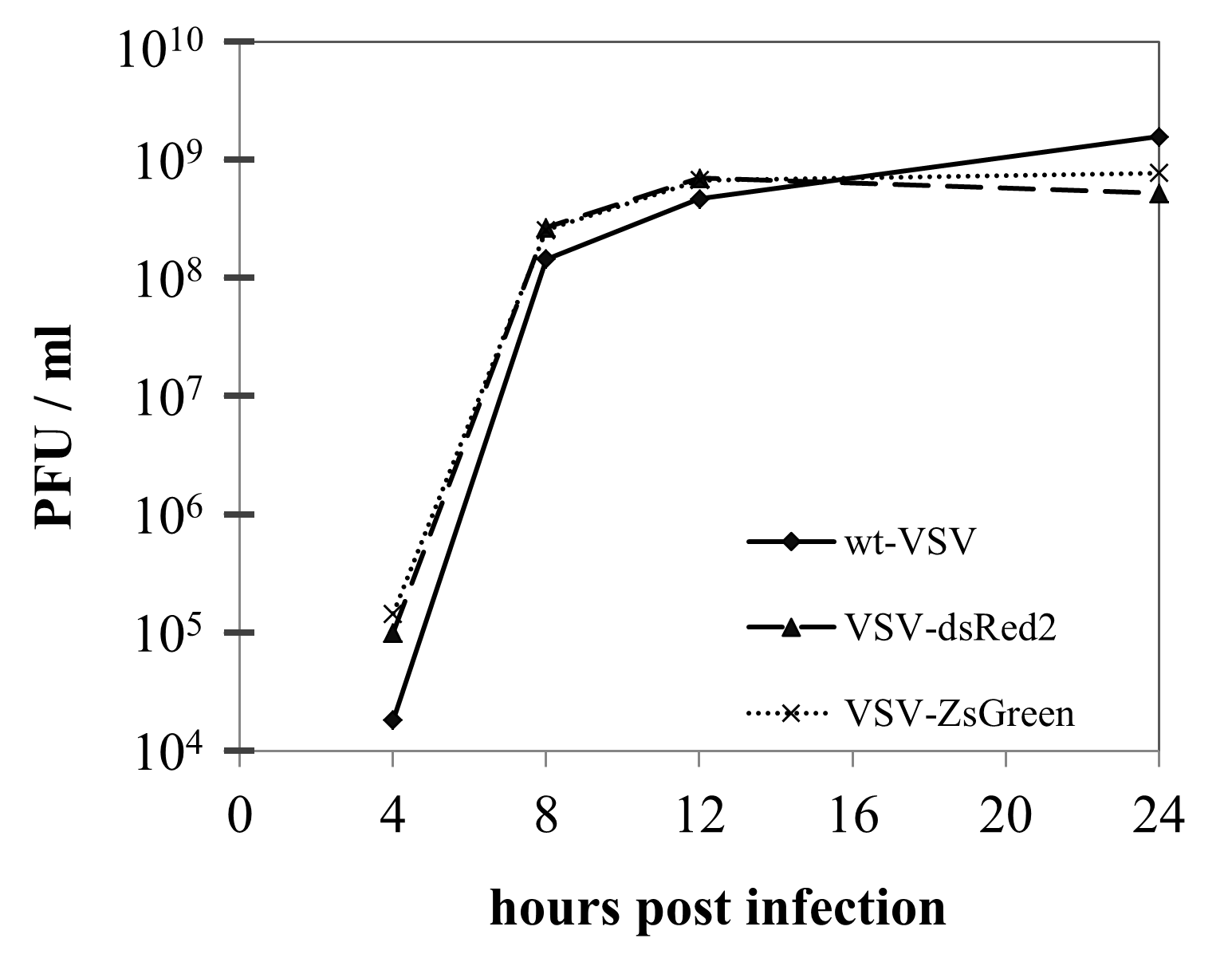

Supplement: Additional file 1: Figure S1 — Kinetics of VSV strain growth on A549 cells. A549 cells were infected in parallel wells, MOI = 10, and parallel supernatant samples were taken over time and titered by plaque assay. [file 1743-422X-10-224-S1.tiff]
