# Supplementary material for: A quantitative infection assay for human type I, II, and III interferon antiviral activities
Source: Virol J. 2013 Jul 6;10:224. doi: 10.1186/1743-422X-10-224 (PMC3716869; doi:10.1186/1743-422X-10-224)
Supplement: Additional file 2: Table S1 — A brief comparison of several published antiviral assays. [file 1743-422X-10-224-S2.doc]

**Supplementary Table 1: A brief comparison of several published antiviral assays.**

| **Assay** | **Antivirals measured** | **Reporter system** | **Cells** | **Linear range of assay** | **Reproducibility** |
| --- | --- | --- | --- | --- | --- |
| This work | human IFNα  human IFNβ  human IFNγ  human IFNλ1  human IFNλ2  human IFNλ3 | Vesicular stomatitis virus reporter expressing DsRed2 fluorescent protein. | A549 human lung epithelial | 0.5-16 U/ml IFNβ,  2-100 U/ml IFNα  250-8000 pg/ml IFNγ  31.2-1000 pg/ml IFNλ1  62.5-1000 pg/ml IFNλ2  3-390 pg/ml IFNλ3 | Inter-assay CV<6%  Intra-assay CV<6% |
| Berger Rentsch et al. 2011 | human IFNβ  chicken IFNα  canine IFNβ  murine IFNβ  porcine IFNα  human IFNλ1 | Vesicular stomatitis virus replicon lacking replication ability expressing eGFP and firefly luciferase. | NHDF human dermal fibroblasts  DF-1 chicken fibroblasts  K-17 canine osteosarcoma  3T3 murine fibroblasts  PK-15 porcine kidney  Calu-3 human lung adenocarcinoma | Approx. 0.2-4 U/ml human IFNβ  Approx. 0.25-16 ng/ml human IFNλ1 | Not reported |
| Bollati-Fogolin et al. 2005 | murine type I IFN | Murine fibroblast reporter cells that express Cre-recombinase upon Mx promotor stimulation, living a block on eGFP expression | Murine fibroblasts | 20-300 U/ml for mIFNα  10-500 U/ml mIFNβ | Not reported |
| Bürgi et al. 2012 | human IFNα  human IFNβ | WISH reporter cell line – Mx promoter-driven eGFP gene | Human WISH cells (HeLa contaminant) | 5.5-2800 U/ml IFNα, 0.7-350 U/ml IFNβ1a,  0.7-1400 U/ml IFNβ1b | <20% CV |
| Canosi et al. 1996 | human type I IFN | Vero reporter cells with Mx-driven luciferase expression | Vero | 0.5 to 32 U/ml IFNα  1 to 16 U/ml IFNβ | Intra-assay CV<7%  Total CV<9% |
| Kugel et al. 2011 | murine type I IFN  murine type III IFN | WISH human reporter cells with Mx2-driven firefly luciferase expression | Murine embryonic fibroblasts lacking functional IFNβ genes. | 1-50 U/ml IFNα  1-12.5 U/ml IFNλ3 | Not reported |
| Kuri et al. 2010 | human IFNα  murine IFNα  chicken IFN | Rift Valley fever virus reporter (BSL3) expressing *Renilla* luciferase | A549 human lung epithelial  L929 murine fibroblasts  Chicken embryo fibroblasts | 1-50 U/ml human IFNα  1-50 U/ml murine IFNα  1-25 U/ml chicken IFN | Not reported |
| Laroque et al. 2011 | human IFNα  human IFNβ | HEK293 reporter cell line – ISRE driven luciferase | HEK293 human embryonic kidney cells | 1.5-50IU/ml IFNα  6.25-200 U/ml IFNβ | <20% CV |
| Lewis 1995 | murine IFNγ  murine IFNβ | Murine GBP promoter driving a bacterial CAT gene in conjunction with a liquid scintillation CAT assay | Murine Ltk-aprt-cells | 5-50 U/mL | Not reported |
